# MYC-Mediated Functional Manifestation of IDH1 Mutations in Intrahepatic Cholangiocarcinoma Confers Sensitivity to (+)-JQ1

**DOI:** 10.7150/ijbs.123085

**Published:** 2026-01-30

**Authors:** Fangyanni Wang, Xinyu Liu, Ning Zhang, Ruirui Kong

**Affiliations:** 1 Translational Cancer Research Center, Peking University First Hospital, Beijing 100034, China.; 2 School of Basic Medical Sciences, International Cancer Institute, Peking University Health Science Center, Beijing 100191, China.; 3 Yunnan Baiyao Group, Kunming 650500, China.

**Keywords:** IDH mutation, MYC, ICC, glutaminolysis, (+)-JQ1

## Abstract

Intrahepatic cholangiocarcinoma (ICC) is one of most aggressive malignancies attributable to limited treatment options. IDH1 is commonly mutated and frequently cooccurred with other genetic alterations in ICC. The mechanism by which they affect ICC patient prognosis and therapeutic resistance remains incompletely understood. We aimed to investigate the function of MYC in IDH1mutant ICC progression and develop the novel therapeutic strategies. We well-established spontaneous ICC mouse models using transposon-based Idh1 and Kras mutations system in liver-specific knockout Trp53 mice. We generated multiple independent primary ICC cells and organoids derived from tumor tissues and established subcutaneous allograft ICC tumors. Together, multiple models in our studies were utilized to elucidate the role of MYC in IDH1-mutant ICC progression and to investigate therapeutic strategies. We demonstrated that the IDH1 mutations correlated with a favorable outcome in ICC patients and murine models. However, MYC overexpression drove the malignant phenotypic manifestation of Idh1 mutations, reversing this phenotype. Mechanistically, Idh1-mutant ICC reprogrammed glutamine metabolism to regulate Myc expression. Notably, ICC with concurrent IDH1 mutations and MYC amplification exhibited sensitivity to the MYC inhibitor (+)-JQ1, but remained resistant to the IDH1 mutation inhibitor AG120. This study identified MYC as a critical pathogenic driver of malignant progression in IDH1-mutant ICC. MYC overexpression conferred resistance to IDH1 mutation inhibitor, while creating a therapeutic vulnerability to MYC-targeted agents. The selective efficacy of (+)-JQ1 against IDH1-mutant ICC identified MYC inhibition as a promising precision medicine strategy for this molecular subset.

## Introduction

Intrahepatic cholangiocarcinoma (ICC) represents the second most common pathological subtype of primary liver cancer (PLC), accounting for 10~15% of all liver malignancies 1,2. Notably, global ICC incidence and mortality rates have shown a rising trend 3,4, whereas the 5-year overall survival (OS) rate for ICC remains dismal at just 9% 5. This poor prognosis is largely due to lack of effective treatment options, highlighting the urgent need for developing novel therapeutic strategies targeting ICC.

Comprehensive genomic profiling has revealed recurrent driver gene alterations in ICC (TP53, KRAS, IDH1 and FGFR2), which affect both cancer progression and treatment response. IDH1 mutations found in 12-30% of cases across cohorts 6,7, and frequently co-occur with KRAS mutations. This co-mutation drives tumorigenesis through synergistic metabolic and epigenetic reprogramming. Mutant IDH1 produces the oncometabolite D-2-hydroxyglutarate (D-2HG), inhibiting α-ketoglutarate (α-KG)-dependent dioxygenases 8-10 and causing epigenetic dysregulation via DNA hypermethylation and altered histone methylation 11-15. Concurrently, mutant KRAS constitutively activates MAPK/ERK and PI3K/AKT/mTOR signaling, stabilizing MYC and upregulating glycolytic and glutaminolytic genes 16. The interplay between IDH1-mediated epigenetic remodeling and KRAS-driven signaling underlies the aggressiveness of co-mutated ICC. These molecular insights have promoted targeted therapy development, particularly for IDH-mutant subtypes.

Despite this progress, clinical outcomes with IDH1 mutation inhibitors remain limited. The phase 3 ClarIDHy trial 17 showed only marginal improvement in median overall survival with ivosidenib versus placebo, prompting exploration of alternative strategies targeting IDH-mutant metabolic vulnerabilities. Emerging evidence reveals tissue-specific vulnerabilities in IDH1/2-mutant tumors. IDH1-mutant AML cells show lipid dependency, requiring de novo fatty acid synthesis for proliferation 18. IDH1-mutant glioblastomas exhibit hypermethylation-mediated suppression of glycolytic genes 19; and IDH1-mutant glioblastoma models and chondrosarcomas display increased sensitivity to glutaminase inhibition 20-22. This spectrum of dependencies, coupled with limited clinical efficacy of direct IDH mutation inhibitors, motivates our investigation of glutamine metabolism as a targetable pathway in IDH1-mutant ICC.

In this study, we established orthotopic ICC mouse models by hydrodynamic delivery of transposon plasmids co-expressing KRAS^G12D^ and IDH1^R132C^ into liver-specific Trp53 knockout mice, which recapitulated the genetic and pathological features of human ICC. This model revealed that MYC overexpression drove malignant progression in IDH1-mutant ICC, leading to unfavorable prognosis. Using a multi-platform approach incorporating derivative primary cells, ICC-derived organoids and syngeneic allografts, we demonstrated that IDH1-mutant ICC reprograms glutamine metabolism to maintain MYC expression and promote cell proliferation. MYC amplification exhibited selective sensitivity to MYC inhibitor (+)-JQ1, while remaining refractory to IDH1 mutation inhibitor AG120, which identified MYC dependency as a therapeutic vulnerability in IDH1-mutant ICC.

## Materials and Methods

### Cell culture

Cholangiocarcinoma cell lines, including one IDH1 mutated (RBE (RRID: CVCL_4896)) and two IDH1/2 wild-type (HuCCT1 (RRID: CVCL_0324) and HCCC9810 (RRID: CVCL_6908)) lines, were analyzed in this study. Cells were cultured at 37°C with 5% CO2 using RPMI 1640 medium (Corning), supplemented with 10% heat-inactivated fetal bovine serum (FBS) (VISTECH) and 1% penicillin/streptomycin (Cytiva). Before experimentation, the authenticity of the cell lines was validated through STR profiling, and mycoplasma contamination was assessed using RT-PCR. Cell lines were maintained for a maximum of three months and were regularly screened for mycoplasma contamination every four weeks to ensure experimental integrity.

### Cell viability and proliferation assay

Cell viability and growth assays were conducted using Cell Counting Kit-8 (Tiangen). Cells were plated at a density of 1000-3000 cells/well in a 96-well plate using glutamine-free RPMI 1640 (Gibco) with 10% FBS and 1% penicillin/streptomycin. After 24 hours, the media was replaced with the appropriate medium with or without 2 mM glutamine. Concurrently, compounds (BPTES, AG120, (+)-JQ1) or DMSO were added at the corresponding concentrations. This time point was defined as initiation at 0 hours. The medium containing 10% CCK8 was replaced at specified intervals, and absorbance at a wavelength of 450nm was measured after a 2-hour incubation period. To assess the drug response curve, the drug was incubated for 48 hours with an initial cell density of 3000 cells per well. The growth curve was generally detected on days 0, 1, 3, 5, and 7, with an initial cell density of 1000 cells per well.

### Plasmid transfection

HuCCT1 cell line was transfected with pcDNA-3.1 plasmid overexpressing wild-type or mutated IDH1 utilizing Lipofectamine 2000 (Thermo Fisher Scientific). Following a 6-hour transfection period, the cells were seeded for cell viability assays, followed by harvesting after 48 hours for qPCR and Western blot analysis.

### Enzymatic measurement of (R)-2HG and α-KG

Tumor samples (10 mg) were collected from pTKI-R132C mice immediately after euthanasia and snap-frozen before storage at -80°C. After digestion, cells were counted, and tissue samples or cells were homogenized in ice-cold RIPA Lysis Buffer (Tiangen). Samples were centrifuged at 13,000 × g for 10 minutes to remove insoluble material. All samples were deproteinized before analysis using Trichloroacetic acid (TCA) precipitation protocols to remove interfering proteins from the sample. Enzymatic measurements were performed using D-2-Hydroxyglutarate (D2HG) Assay Kit (Sigma, catalog no. MAK320) and α-Ketoglutarate Assay Kit (Sigma, catalog no. MAK054) following the manufacturer's protocol.

### RNA isolation and qPCR

Total RNA was isolated utilizing Trizol and chloroform. To initiate the process, 500 μL of Trizol was added to the cell pellet, followed by resuspension and a 10-minute incubation at 4 ℃. Subsequently, 100 μL of chloroform was added, the mixture was centrifuged at maximum speed for 15 minutes, resulting in the formation of three distinct layers. The top aqueous layer was then transferred to a new tube and mixed with an equal volume of isopropanol to precipitate the RNA. After a 10-minute room temperature incubation, the tube was centrifuged at maximum speed for 10 minutes, leading to the formation of a pellet. The pellet was washed twice with 70% ethanol before being dissolved in molecular-grade water. An amount of 2 μg RNA was used to synthesize cDNA through reverse transcription using the FastKing RT Kit (Tiangen). Subsequently, quantitative real-time PCR (qPCR) analysis was conducted utilizing the SuperReal PreMix Plus (SYBR Green) (Tiangen) on an AriaMx real-time PCR system (Agilent). The relative gene expression levels were determined and normalized utilizing the 2ΔΔCt method with the primers in Supplementary Table 1.

### RNA sequencing and data analysis

Total RNA was isolated from the sample using TRIzol Reagent following the manufacturer's instructions. The quality and concentration of the RNA were evaluated using a 5300 Bioanalyzer (Agilent) and an ND-2000 spectrophotometer (NanoDrop Technologies), respectively. We selected only high-quality RNA samples for sequencing, defined by specific criteria: OD260/280 ratios between 1.8 and 2.2, OD260/230 ratios of at least 2.0, RQN values of 6.5 or higher, 28S:18S ratios of at least 1.0, and concentrations exceeding 1 µg. The RNA samples underwent purification, reverse transcription, library construction, and sequencing at Shanghai Majorbio Bio-pharm Biotechnology Co., Ltd. in Shanghai, China. For library construction, we used 1 µg of total RNA with the Illumina Stranded mRNA Prep Ligation Kit. The libraries were prepared by selecting cDNA fragments of 300 bp on a 2% Low Range Ultra Agarose gel, followed by 15 cycles of PCR amplification using Phusion DNA polymerase (NEB). After PCR, the libraries were quantified with a Qubit 4.0 fluorometer. Sequencing was performed on a NovaSeq X Plus platform using the NovaSeq Reagent Kit (PE150 mode).

The RNA-seq reads were aligned to the human reference genome, GRCh38.p13, utilizing HiSat2 and gene expression levels were quantified with RSEM. Differentially expressed genes (DEGs) were identified using DESeq2, with a fold change threshold of 2 or more and an adjusted p-value below 0.05. Pathway enrichment analysis was carried out using the R package clusterProfiler.

To investigate a comprehensive, systems-level view of the gene interactions that define the metabolic landscape of IDH1-mutant ICC, we first identified common targets by intersecting the significantly upregulated genes with the targets of metabolic pathways (hsa01100) using a Venn diagram. These common targets were then imported into the STRING11.0 database (https://string-db.org/) for network topological analysis, with a combined score threshold of > 0.7. The interaction network was visualized using Cytoscape software (Version 3.6.0).

### Western blotting

Cells were lysed using RIPA buffer supplemented with protease and phosphatase inhibitors (Roche). Protein concentrations were measured using the BCA assay (Thermo Scientific). Proteins were then resolved on 10% SDS-polyacrylamide gels and transferred onto Immuno-Blot PVDF membranes (Bio-Rad). Following the transfer, membranes were blocked for one hour at room temperature using 5% milk powder in TBS-T (0.1% Tween). Overnight incubation at 4°C with primary antibodies followed, specifically anti-c-Myc (Abcam, ab32072) and anti-α-Tublin (Proteintech, 14555-1-AP) or anti-Vinculin (Proteintech, 26520-1-AP). Membranes were washed three times with TBS-T and then incubated with HRP-conjugated secondary antibodies (anti-rabbit or anti-mouse IgG, CST, 7074 & 7076) for one hour at room temperature. Blots were visualized using enhanced chemiluminescence (Thermo Scientific).

### Mice

All mouse experiments were approved by the Animal Care and Use Committee at Peking University Health Science Center (Approval No. #PUIRB-LA2022626). The mice were housed in a pathogen-free environment with ambient temperatures ranging from 20-26 °C, humidity levels maintained between 30%-70%, and subjected to a 12:12 h light-dark cycle, as specified by the animal facility protocols at Peking University Health Science Center. Before the experiments, all mice underwent health checks and acclimation to the laboratory environment to ensure they were in optimal condition.

Trp53^fl/fl^ and Alb-Cre mice with a C57BL/6 background were procured from Jackson Laboratory. Trp53^fl/fl^ mice were crossed with Alb-Cre mice to generate liver conditional Trp53 knockout (Trp53 cKO) mice. Sleeping beauty transposase (SB100), transposon pT3-Neo-EF1a-GFP plasmids and pSpCas9(BB)-2A-GFP (PX458) plasmid were sourced from Addgene.

Kras^G12D^ and wild-type IDH1 or IDH1^R132C^ fragments were amplified through PCR cloning of mouse cDNA. Kras^G12D^ was integrated into the transposon vector using the MluI and SpeI restriction enzyme sites, while IDH1 fragments were inserted at AscI and NotI sites, resulting in the development of the Kras^G12D^ and IDH1^wt/R132C^ transposon plasmids (pT3-Kras^G12D^-IDH1^wt/R132C^, pTKI-wt/R132C). Subsequently, the cDNA of the mouse Myc gene was cloned into the transposon vector via the MluI and SpeI sites, resulting in the generation of the pT3-Neo-EF1a-Myc plasmid (pTM). Plasmids for hydrodynamic tail vein (HDTV) injection were prepared using an EndoFree-Maxi Kit (Qiagen).

A DNA mixture containing 25 μg of transposon and 5 μg of transposase-encoding plasmid was suspended in 0.9% saline solution for HDTV injection. The total volume, equivalent to 10% of the body weight of 8-week-old male Trp53 cKO mice, was injected into the tail vein within 5-7 seconds. An additional 10 μg of pTM plasmid and 2 μg of transposase-encoding plasmid were included if required. For wild-type mice, 14 μg of PX458-sgTrp53 plasmid was essential. After anesthetization, all mice exhibited palpable liver tumor nodules. The median survival time was recorded as previously stated for each mouse model.

Twenty days after injection, mice injected with pTKI+pTM were randomly assigned to different groups. To evaluate the efficacy of drug treatments in inhibiting tumor growth in situ, the mice were treated daily with either (+)-JQ1 (50 mg/kg, intraperitoneal) or AG120 (100 mg/kg, oral gavage) until their demise. The effectiveness of these treatments was measured by analyzing survival times and tumor weights.

### Histopathological and immunohistochemistry (IHC) analysis

Formalin-fixed, paraffin-embedded liver tissue sections (4 μm thick) were prepared for histological evaluation in a mouse tumor model. Hematoxylin and eosin (H&E) staining was performed on each section. Alternatively, the sections were stained with Sirius Red in identification of collagen deposit in liver tumor. Two board-certified renal pathologists independently evaluated histopathological alterations in a blinded manner. For immunohistochemistry (IHC), sections were deparaffinized in xylene and rehydrated through a graded ethanol series (100%, 95%, and 70%). Antigen retrieval was achieved by microwaving the slides in a citric acid solution for 15 minutes. The primary antibodies are incubated overnight at the dilution of 1:200. Sections were then incubated with HRP-conjugated secondary antibodies at a 1:1 ratio (100 μL per slide) for 10 minutes at room temperature. The digital microscope camera system 3DHISTECH was used to capture images of each part. Antibodies used for IHC are listed in Supplementary Table 2.

### Tumor tissue dissociation and culturing

The mice were euthanized, and their tumors were dissected. Tumor tissue from the PTKI-R132C mouse model was finely minced into small pieces (approximately 0.5-1 mm in diameter) using delicate dissection scissors. Subsequently, the tissue pieces were rinsed three times with phosphate-buffered saline. The minced tissue was then dissociated using tumor dissociation medium, consisting of Advanced DMEM/F12 (Gibco) supplemented with 1% penicillin/streptomycin (Thermo Fisher Scientific), 0.1 mg/mL collagenase A (Sigma), 0.1 mg/mL Dispase II (Sigma), 0.05 mg/mL DNase I (Roche), and 1:100 insulin-transferrin-selenium-ethanolamine (Thermo Fisher Scientific). The dissociation process involved incubating at 37℃ for 20-30 minutes until most cells were suspended. The suspended cells were then centrifuged at 400 × g for 5 minutes. After removing the supernatant, the cell pellets were resuspended and incubated in 1X RBC lysis buffer (Tiangen), rotating gently for 5 minutes at room temperature to lyse any contaminating red blood cells. Following another round of centrifugation, the cells were treated with TrypLE (Gibco) for 10 minutes at 37℃. The digestion process was stopped by adding cold DMEM supplemented with 10% FBS. The cells were then filtered through a 70 μm Nylon cell strainer and centrifuged for 5 minutes at 400 × g. Finally, the cells were cultured utilizing various methods to generate primary cells or organoids.

Cells were encapsulated in Matrigel (Corning) for organoid culture and dispensed as droplets into ultralow-attachment 6-well plates, based on viable cell counts. The Matrigel solidified within 30 minutes, after which warm organoid isolation culture medium was added. This medium included Advanced DMEM/F12, 1% penicillin/streptomycin, 1% GlutaMAX, 10 mM HEPES, 1:50 B27 supplement, 1:100 N2 supplement, 80 μM N-acetyl-L-cysteine, 5 μM A83-01, and recombinant growth factors (50 ng/mL EGF, 100 ng/mL FGF10, 25 ng/mL HGF). The medium was refreshed every 3-4 days. Organoids were passaged weekly at a 1:2-4 dilution using mechanical dissociation with TrypLE, reducing them to single cells. Organoids were also dissociated into single cells to create frozen stocks, which were stored in 90% FBS (VISTECH) and 10% DMSO (Sigma) at -80°C.

Organoid cells (8000/well in 100 μL isolation culture medium supplemented with 5% Matrigel and without glutamax) were plated into low-attachment 96-well plates. After 24 hours, GlutaMax and the compounds AG120 and (+)-JQ1, or DMSO as control, were added at specified concentrations. Following a 5-day incubation with the compounds, cytotoxicity was assessed using the CellTiter-Glo Luminescent Cell Viability Assay (Vazyme) and a microplate reader.

The primary tumor cells were resuspended and grown in RPMI 1640 medium supplemented with 1% penicillin/streptomycin, 10% FBS, and 50 ng/mL recombinant mouse EGF. Upon adhering to the surface and displaying rapid growth, the cells were then maintained following a standard culture protocol similar to the aforementioned cell lines.

### Establishment of Myc knockout cell lines

To construct the Myc knockout plasmid, a single-guide RNA (sgRNA) with the sequence 5'-CACCgGGACGTAGCGACCGCAACAT-3' was inserted into the pSpCas9(BB)-2A-GFP (PX458) plasmid via homologous recombination. The recombinant plasmid PX458-sgMyc was transfected into primary tumor cells with Lipofectamine 2000 (Thermo Fisher Scientific). Then green fluorescence-positive cells were isolated via a SymphonyS6 flow cytometer (BD) according to the manufacturer's instructions. The isolated single cell was cultured in 96 plates following a standard culture protocol similar to the aforementioned cells. The expression of Myc in edited cells was detected via western blotting. The primary tumor cells which were transfected with PX458 empty plasmid, was applied as a control. Subsequent experiments were performed after the cells had been passed on for 2-3 times.

### Cell derived xenograft (CDX)

For subcutaneous grafting, tumor cells were cultured, dissociated into single-cell suspensions using 0.25% trypsin (GIBCO), and washed twice with PBS. Cells were then suspended in a 1:1 mixture of DMEM (Corning) and growth factor-reduced Matrigel (Corning). Subsequently, 4 × 10^6^ ICC cells were injected into six-week-old male C57BL/6J mice for the subcutaneous models. Ten days after injection, mice were randomly divided into groups when the tumor volumes reached approximately 200 mm^3^ (n = 5). To assess the efficacy of drug treatment in inhibiting tumors in situ, the mice received a daily intraperitoneal injection of (+)-JQ1 (50 mg/kg, intraperitoneal) or AG120 (100 mg/kg, oral gavage) for the following 2 weeks. Tumor volume was measured and quantified every four days using the formula: tumor volume = ½ length × width2. The mice were euthanized after four weeks.

### Statistical analysis

Data were analyzed utilizing the GraphPad Prism 9.5.1 software and presented as means ± SD. Unless specified differently, p-values for comparing two means were calculated utilizing the two-tailed unpaired Student t-test. IC_50_ values were determined utilizing the dose-response (inhibition) function in Prism. Kaplan-Meier method and log-rank test were used for survival analysis. Results are presented as mean ± standard deviation (SD) from at least three replicates. p values<0.05 were considered statistically significant.

## Results

### IDH1-mutant ICC patients and mouse models exhibit a better prognosis than those with wt IDH1

**Figure 1:**
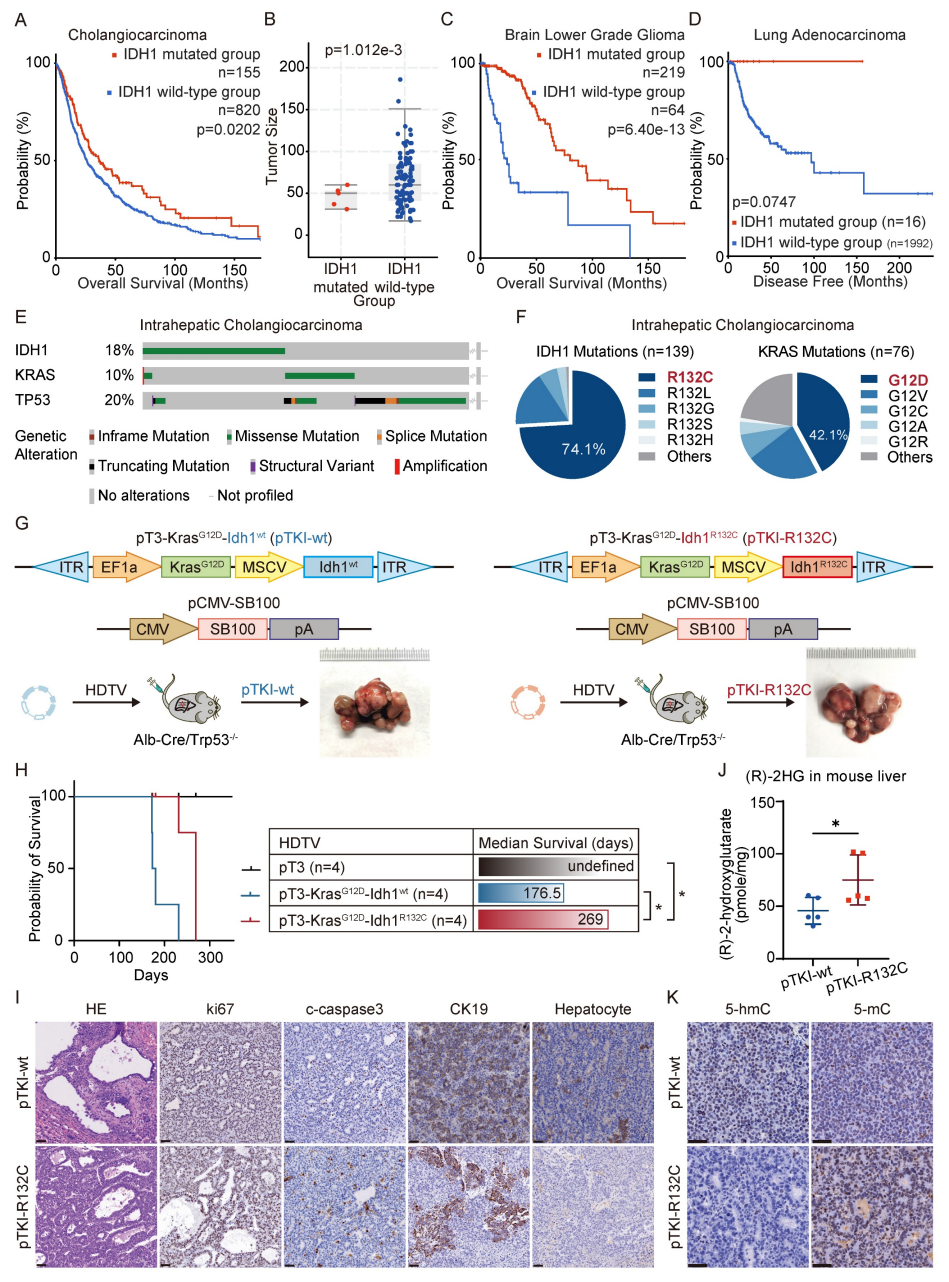
** IDH1-mutant ICC patients and mouse models exhibit a better prognosis than those with wild-type IDH1.** A. Overall survival in cholangiocarcinoma patients with IDH1 mutations and wild-type patients in the clinical cohort. Kaplan-Meier curves were analyzed using the log-rank. B. Tumor size in intrahepatic cholangiocarcinoma patients with IDH1 mutations and wild-type patients in the clinical cohort. C. Overall survival in brain lower grade glioma patients with IDH1 mutations and wild-type patients in the clinical cohort. Kaplan-Meier curves were analyzed using the log-rank. D. Overall survival in lung adenocarcinoma patients with IDH1 mutations and wild-type patients in the clinical cohort. Kaplan-Meier curves were analyzed using the log-rank. E. IDH1, KRAS, and TP53 mutation frequency in human ICC (data obtained from cBioprotal). F. Relative frequency of IDH1-mutant variants in human ICC (data obtained from cBioprotal). G. Schematic and representative macroscopic tumor tissues of the mouse models. The transposable vectors pTKI-wt (encoding Idh1^wt^ and Kras^G12D^, left) or pTKI-R132C (encoding Idh1^R132C^ and Kras^G12D^, right) plus the SB100 vector for transposase expression were delivered into Alb-Cre × Trp53^fl/fl^ mice via HDTVi. Rulers in the photo show a minimum unit of mm. H. Survival graph of the pT3 (control), pTKI-wt/Trp53^-/-^ and pTKI-R132C/Trp53^-/-^ mice. A log-rank test was used to calculate p-values. n=4. *, p < 0.05. J. Concentration of (R)-2HG in livers from mice with the indicated genotypes detected by (R)-2HG assay kit. n=5. *, p < 0.05. I. Representative hematoxylin and eosin (H&E) and IHC staining of Ki67, cleaved-caspase3, HCC marker hepatocyte and ICC marker CK19 from mouse liver tissues. Scale bar: 50 μm. K. Representative IHC staining of 5-hmC and 5-mC from mouse liver tissues. Scale bar: 50 μm.

Emerging evidence established IDH1 mutations as clinically significant molecular alterations across multiple malignancies, including their prognostic implications. Our integrated analysis of cholangiocarcinoma clinical cohorts demonstrates that IDH1 mutations were significantly associated with improved overall survival (Fig. 1A) and reduced tumor burden compared to IDH1-wildtype cases (Fig. 1B). Consistently, clinical cohort data indicate that IDH1 mutations positively correlated with superior survival outcomes of low-grade glioma (LGG) and lung adenocarcinoma (LUAD) (Fig. 1C-D). To further investigate the function of IDH1 mutations in ICC tumorigenesis and prognosis, we developed isogenic mouse models with mutant IDH1 or wildtype IDH1. Informed by the FU-iCCA cohort's mutational landscape 6, TP53 and KRAS mutations are among the most frequently altered genes in ICC, alongside IDH1and BAP1 mutations and FGFR2 fusions. Furthermore, we found that genetic alterations in TP53, KRAS, and IDH1, but not BAP1 or FGFR2, were associated with worse prognosis compared to their genetic un-alteration counterparts respectively (Fig. S1A), suggesting these three genes may have cooperative role to provoke the pro-tumor phenotype. Therefore, we focused on TP53, KRAS, and IDH1 as ICC drivers for modeling. Genomic analysis of 782 ICC cases across five independent cohorts 23-27 (via cBioPortal) identified the following mutation frequencies: IDH1 (18%, 139/782 cases), TP53 (20%), and KRAS (10%) (Fig. 1E). Among IDH1 mutations, 74% (103/139) occurred at the canonical R132 hotspot, predominantly R132C substitutions. Similarly, KRAS mutations primarily affected codon 12, with G12D being the most common mutation (Fig. 1F).

Leveraging clinical mutation frequencies, we constructed transposon vectors expressing murine Kras^G12D^ (driven by weak EF1α promoter) and Idh1^R132C^ (under strong MSCV promoter). This vector was co-delivered via hydrodynamic injection with Sleeping Beauty SB100 transposase into the hepatocytes of conditional liver Trp53 knockout mice (Albumin Cre; Trp53^fl/fl^) to generate tumors, as referred to pTKI-R132C. We also established pTKI-wt tumor models with transposon-mediated murine Kras^G12D^ and wild type Idh1 system in liver specific Trp53 KO mice (Fig. 1G). Additionally, we injected with equal control plasmid (pT3) to establish a negative control. Both pTKI-R132C and pTKI-wt mice developed multifocal liver tumors with 100% penetrance. Strikingly, pTKI-R132C mice exhibited significantly prolonged median survival (269 days) versus pTKI-wt controls (176.5 days), consistent with the improved prognosis observed in IDH1-mutant ICC patients (Fig. 1H). Immunohistochemical analysis revealed markedly elevated proliferation indices (Ki67^+^ cells) and apoptotic activity (cleaved caspase-3^+^), indicating that pTKI-R132C and pTKI-wt provoked aggressive tumors.

Furthermore, we comprehensively analyze the histopathological feature of tumor tissues from both genotypes. Both tumors displayed highly differentiated glandular architecture and stromal invasion on H&E staining, along with uniform expression of the biliary lineage marker cytokeratin 19 (CK19) and absence of the hepatocellular marker hepatocyte, confirming the histological classification as ICC (Fig. 1I). Stromal organization, assessed via α-SMA immunohistochemistry and Sirius Red staining, showed comparable patterns of cancer-associated fibroblast distribution and collagen deposition. Immune cell infiltration, evaluated by CD45^+^ staining, displayed focal and extensive patterns in both genotypes without statistically significant variation in density and spatial distribution (Fig. S1B-C). These findings validated that our pTKI-R132C and pTKI-wt models recapitulated histopathological features of human ICC.

Metabolomic analysis revealed significantly elevated 2-hydroxyglutarate (2-HG) levels in pTKI-R132C tumors compared to pTKI-wt controls (Fig. 1J). Furthermore, immunohistochemistry demonstrated that IDH1-mutant tumors exhibited markedly reduced 5-hydroxymethylcytosine (5hmC) levels and concurrent increases in 5-methylcytosine, compared to those with wild-type IDH1 (5mC) (Fig. 1K). These findings in the murine model corroborated previous observations in ICC patients, suggesting that IDH1 mutation impedes the function of the TET family of DNA dioxygenases, resulting in reduced cytosine hydroxymethylation alongside an increase in DNA methylation 13. These results confirmed that the Idh1^R132C^ allele showed stable expression and maintained enzymatic activity in tumor tissue.

In order to implement an easy-to use ICC model, we utilized the combination of CRISPR-Cas9 and transposon-based strategy in vivo to target the DNA of hepatocytes with HDTVi, capable of editing genes and integrating into genomics. We designed specific guide RNAs against to target the first exon of murine Trp53 (sgTrp53) to generate CRISPR-sgTrp53 plasmids, as described in Figure S2. Then, pTKI-R132C plasmids and CRISPR-sgTrp53 were co-administered into wild-type mice. As respected, all pTKI-R132C/sgTrp53 mice developed tumors similar to pTKI-R132C / Trp53^-/-^mice. Taken together, these data suggested that Kras^G12D^ and Idh1^R132C^ mutations as well as loss-of-function of Trp53 mediated by either the Cre-LoxP or CRISPR-Cas9 system could induce carcinogenesis in liver.

In conclusion, we found that mutant IDH1 predicted better prognosis than wild-type IDH1 for ICC patients and our established ICC mouse models.

### Cooperation of MYC with IDH1 mutation drives a worse survival

IDH1 mutation generates oncometabolite R-2HG, which can be not used by any primary metabolic pathways and instead accumulates to high levels in the cell, altered enzyme activity and disrupted epigenetic and metabolic regulation, leading to widespread biological dysregulation 11,12. We hypothesized that the improved prognosis associated with IDH1 mutations in certain solid tumors (e.g., ICC, gliomas) may reflect incomplete penetrance, where only a subset of mutation-bearing cells manifested the full oncogenic phenotype.

**Figure 2:**
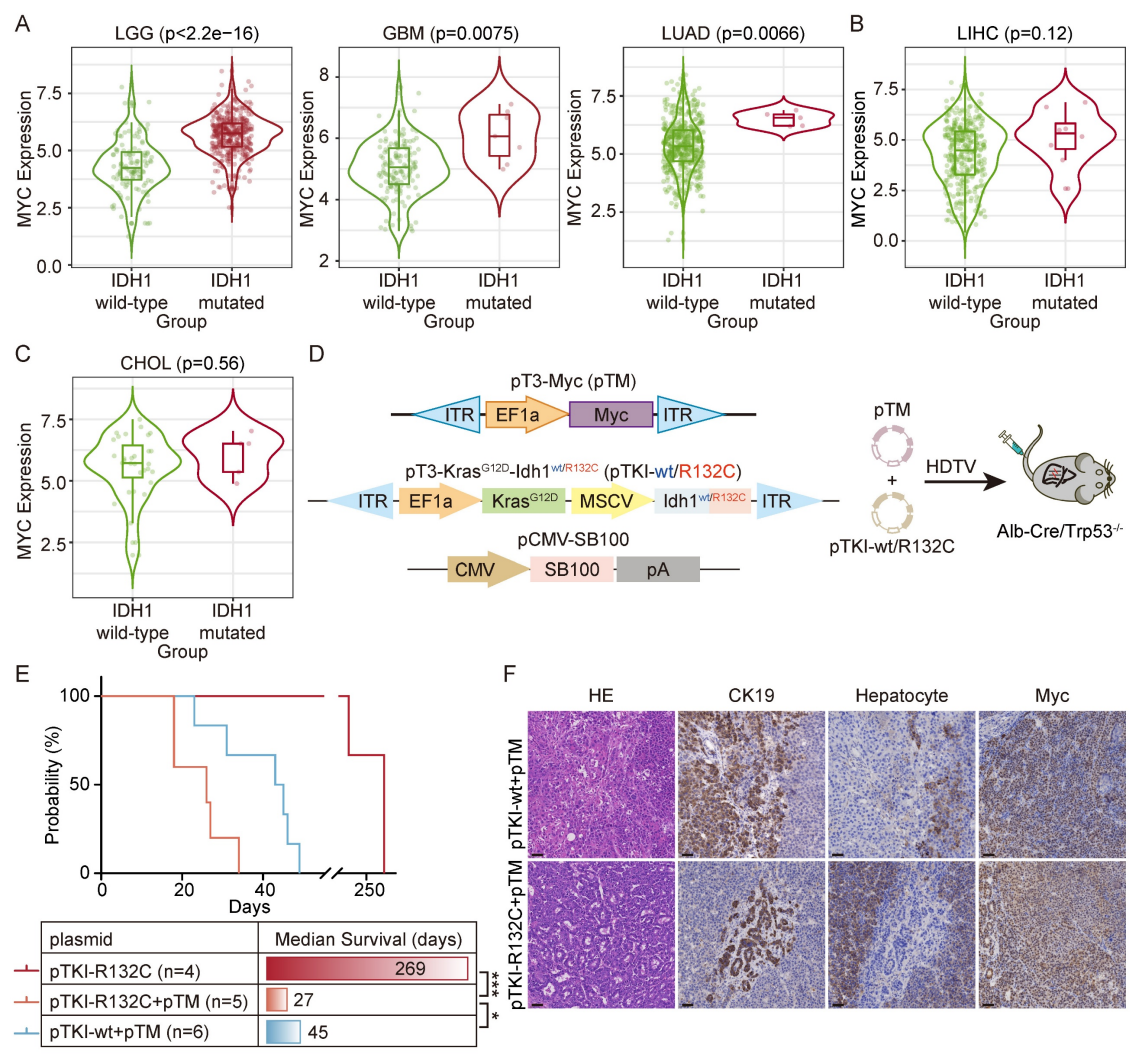
** Cooperation of MYC with IDH1 mutation drives a worse survival.** A-C. In the TIMER2.0 database, it was found that there was a significant correlation between IDH1 mutation and the high expression of MYC in LIHC, LGG, GBM, LIHC and CHOL patients. p-values were calculated using Wilcoxon rank-sum test. D. Schematic of the mouse model. The transposable vector pTM (encoding Myc) with pTKI and SB100 vector was delivered into Alb-Cre × Trp53^fl/fl^ mice via HDTVi. E. Survival graph of the pTKI-R132C and pTKI+pTM mice. A log-rank test was used to calculate p-values. *, p < 0.05. ***, p < 0.001. F. Representative IHC staining from mouse liver tissues. Scale bar: 50 μm.

To search for the genes involved in manifestation of IDH1 mutations in tumors, we analyzed the co-occurrent genes with IDH mutation by the TIMER2.0 database. The results demonstrated that MYC expression was elevated in IDH1 mutant LGG, GBM and LUAD, but not in their wild-type counterparts (Fig. 2A). A similar MYC upregulation trend was also observed in IDH1-mutant liver hepatocellular carcinoma (LIHC) and cholangiocarcinoma (CHOL) (Fig. 2B-C). We therefore hypothesized that there is a synergistic relationship between MYC amplification and IDH1 mutation, and that MYC amplification may increase the penetrance of the IDH1 mutation-driven oncogenic phenotype.

Next, we introduced pTKI-R132C and MYC-expressing transposon vector (known as pTM) into Alb-Cre/Trp53^fl/fl^ mice (Fig. 2D). The results demonstrated that the upregulation of Myc in IDH1-mutant mice dramatically accelerated tumor initiation and progression, leading to a drastically short median survival time to 27 days. Strikingly, both pTKI-wt and pTM-bearing mice exhibited the median survival time of 45 days, significantly surpassing that of the pTKI-R132C+ pTM group (Fig. 2E). These results suggested that Myc had a more substantial tumorigenic influence on Idh1 mutant models.

Immunohistochemical analysis confirmed that all experimental tumors exhibited the glandular architecture and biliary epithelial marker CK19-positive staining (Fig. 2F), demonstrating that MYC overexpression accelerates tumor progression without altering ICC pathological features. These mouse models recapitulated the IDH1-mutant/MYC-amplified ICC, providing a robust platform for preclinical drug evaluation, mechanistic insights into oncogene cooperativity and opportunities for biomarker discovery in this aggressive ICC.

### AG120 demonstrates poor efficacy in IDH1-mutant ICC with high MYC expression

**Figure 3:**
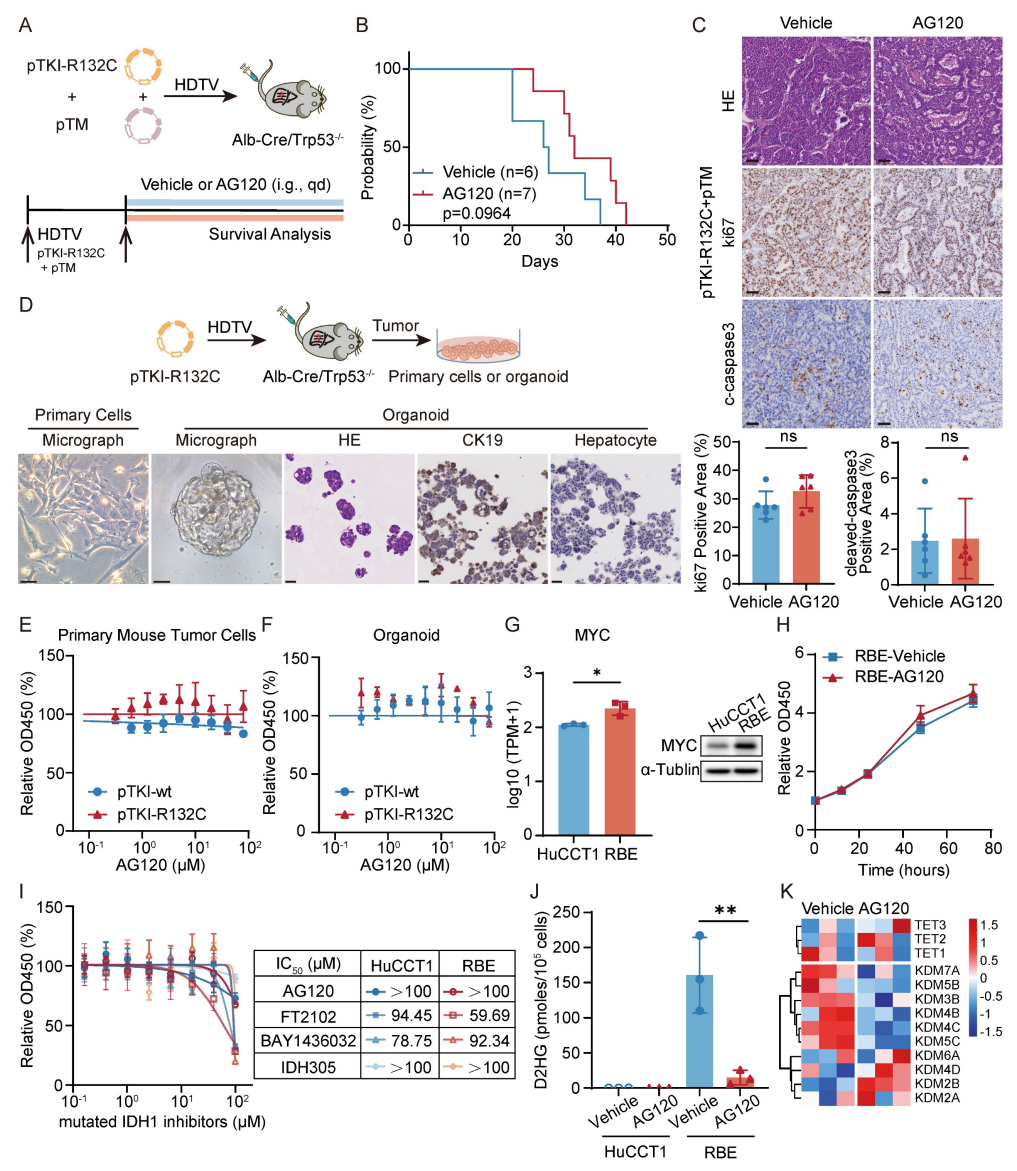
** AG120 demonstrates poor efficacy in IDH1-mutant ICC with high MYC expression.** A. Schematic of the experiment. The transposable vector pTM with pTKI-R132C and SB100 vector was delivered into Alb-Cre × Trp53^fl/fl^ mice via HDTVi. The mice treated with vehicle or AG120. B. Survival graph of the pTKI-R132C+pTM mice with vehicle (n=6) or AG120 treatment (n=7). A log-rank test was used to calculate p-values. C. Representative H&E and IHC images and quantification of area performed by Ki67 and cleaved caspase3 immunohistochemistry on liver tumor tissues of mice treated with vehicle or AG120. Scale bar: 50 μm. ns, p>0.05. D. Schematic of development of the pTKI-R132C organoid and primary tumor cells (top) and representative macroscopic image, H&E and IHC staining of primary tumor cells or organoid (bottom). Scale bar: 50 μm. E-F. Dose-response curves of AG120 for primary tumor cells (E) and organoid (F) derived from pTKI mouse. n=5. G. RNA sequencing and western blot determined differences in MYC gene expression between two cell lines. n=3. *, p < 0.05. H. AG120 did not suppress cell growth in RBE cells. Cell proliferation was examined using the CCK-8 assay. Cell number was normalized to day 0. p-values were calculated using the two-way ANOVA. n=5. I. Dose-response curves of several inhibitors targeting IDH1 mutations for HuCCT1 and RBE. n=5. J. Concentration of (R)-2HG in cell lines under AG120 treatment detected by enzymatic assay kits. n=3. **, p < 0.01. K. Heat maps showed the expression of important genes in 2-HG target TET family and KDM family of RBE cells after 48 hours of treatment with or without AG120 by RNA sequencing.

Clinical trial results indicated that patients with ICC exhibited limited efficacy and can developed resistance to ivosidenib (AG120) 17. Administration of AG120 in our pTM+pTKI-R132C mouse model failed to significantly improve the survival of the mice (Fig. 3A-B). Additionally, no reduction of the proliferation marker ki67 and increase in apoptosis marker cleaved-caspase3 was observed in the tumor tissues of two groups (Fig. 3C). The results demonstrated that AG120 shows no substantial therapeutic efficacy in vivo for intrahepatic cholangiocarcinoma in IDH1-mutant mice with increased Myc expression.

To further validate these phenomena in vitro experiments, we developed organoids and primary cells derived from pTKI^wt/R132C^/Trp53^-/-^ tumor tissues. Under microscopic examination, the organoids exhibited adenoid differentiation or mucous secretion phenotypes, maintaining the key ICC histopathological features (Fig. 3D). Using these primary cells and organoids, AG120 did not exhibit any noticeable cytotoxic effect, consistent with the in vivo findings (Fig. 3E-F).

Furthermore, we also examined the efficacy of AG120 on human ICC cell lines. The study utilized HuCCT1 cell lines with wild-type IDH1, and the RBE cell line harboring an R132S mutation in the IDH1 gene. Initially, the capacity of cell lines to generate 2-HG was assessed, revealing significant 2-HG production in RBE (Fig. S3A). RNA-seq and western blot also revealed a substantial increase in the expression of MYC in RBE cells compared to HuCCT1 cells (Fig. 3G).

To assess the fidelity of these cell lines in representing key tumor characteristics, RNA-seq analysis was performed to compare the gene expression in the cell lines with the signaling pathways notably altered in tumor tissue from ICC patients with IDH1 mutations. Recent research has revealed decreased gene sets related to cytokine and NF-κB signaling and elevated gene sets associated with the FGFR, PI3K, and IRS signaling pathways in cases of intrahepatic cholangiocarcinoma carrying IDH1 or IDH2 mutations 13. Gene Set Enrichment Analysis (GSEA) showed that RBE cells exhibited a notable increase in gene sets associated with FGFR, PI3K, IRS, and other signaling pathways (Fig. S3B). Furthermore, compared with HuCCT1, KEGG pathway enrichment analysis and Reactome pathway enrichment analysis indicated significant down-regulation of pathways such as NFκB signaling pathway, chemokine-related signaling pathway, and IFNγ signaling pathway in RBE cells (Fig. S3C). GSEA also showed that pTKI-R132C mouse tumors exhibited a notable decrease in gene sets associated with IFNγ response pathway compared with pTKI-wt mouse tumors (Fig. S3D). To summarize, the RBE cell line with IDH1 mutation and the HuCCT1 cell line without IDH1 mutation accurately replicated the changes observed in multiple signaling pathways in patient tumor tissues, suggesting their utility as representative research models for further investigations.

The growth kinetics analysis confirmed that AG120 was unable to suppress the proliferation of RBE cells (Fig. 3H). Moreover, multiple inhibitors targeting IDH1 mutations were administered to cell lines, including AG120. The results showed no substantial difference in the dose-response curves of RBE and HuCCT1 to drugs below 100 μM (Fig. 3I). Following detection, AG120 markedly decreased the levels of 2-HG in RBE cells (Fig. 3J). RNA-seq revealed upregulation of parallel epigenetic regulators in the TET and KDM families following 2-HG depletion (Fig. 3K), suggesting that redundant epigenetic regulation and activation of bypass signaling pathways in IDH1-mutant ICC.

Taken together, AG120 failed to suppress proliferation of IDH1-mutant/MYC-amplified ICC cells in vivo and in vitro experiments, indicating that MYC amplification appears to override IDH1 mutation dependency and initiate metabolic bypass pathways to confer intrinsic resistance.

### Glutaminolysis metabolism promotes the growth of IDH1-mutant intrahepatic cholangiocarcinoma cells and the high expression of MYC

**Figure 4:**
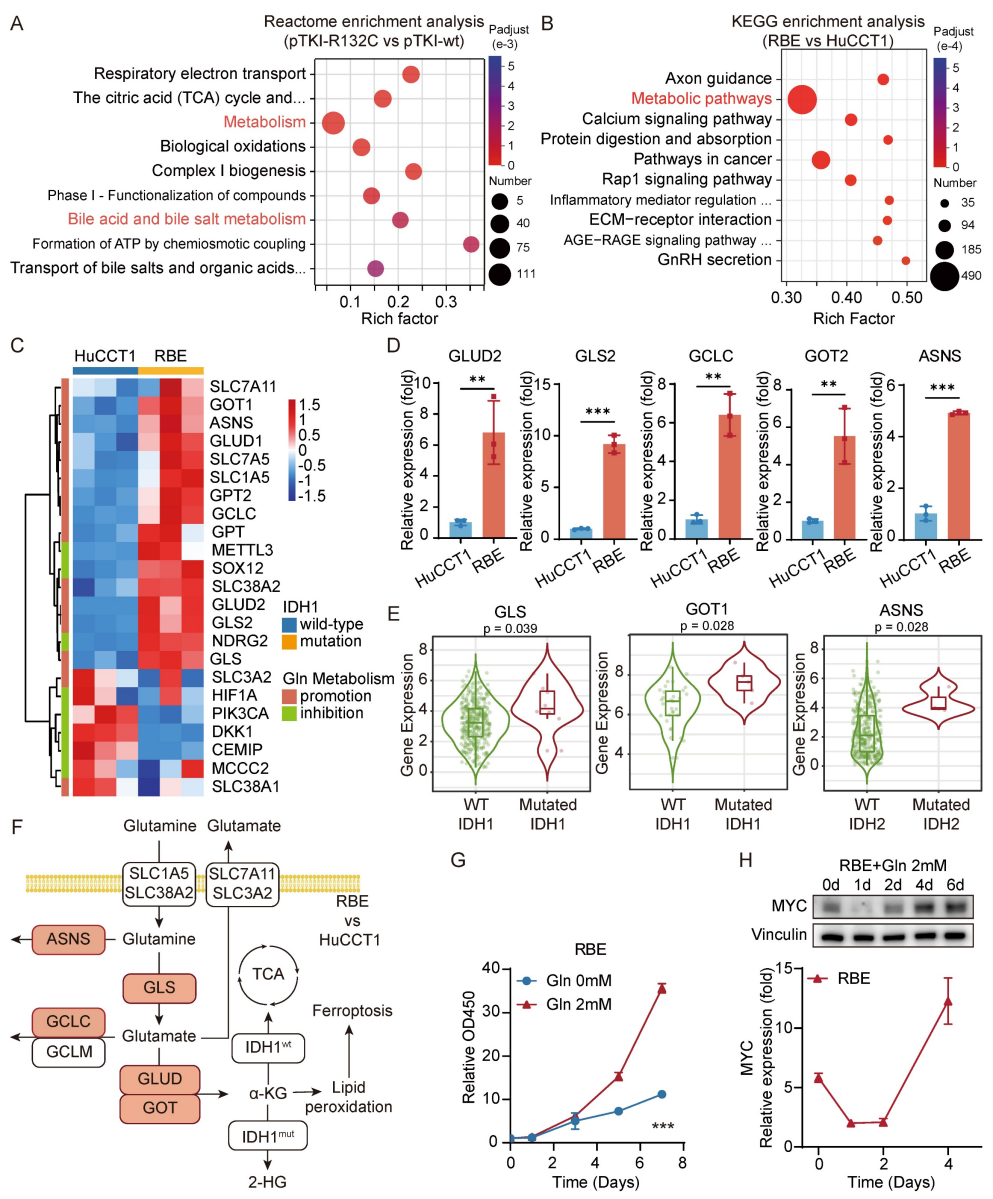
** Glutaminolysis metabolism promotes the growth of IDH1-mutant intrahepatic cholangiocarcinoma cells and the high expression of MYC.** A. Reactome enrichment analysis were performed for genes with significantly differential expression in pTKI-R132C compared with pTKI-wt obtained by RNA-seq. B. KEGG enrichment analysis were performed for genes with significantly differential expression in RBE compared with HuCCT1 obtained by RNA-seq. C. Heat maps showed differences in the expression of important promotion genes and inhibition genes involved in glutaminolysis signaling pathway between HuCCT1 and RBE cell lines. D. The expression difference of GLS2, GOT2, ASNS, GLUD2 and GCLC in HuCCT1 and RBE cells was verified by qRT-PCR. n=3. **, p < 0.01. ***, p < 0.001. E. The expression difference of GLS, GOT1 and ASNS in tumor tissues of CHOL (n=36) or LIHC (n=365) patients with IDH1/2 mutation was analyzed using the TIMER2.0 database. p-values were calculated using Wilcoxon rank-sum test. F. Diagram of the glutamine-related metabolic signaling pathways, with red representing genes up-regulated in RBE compared with HuCCT1. G. 2mM glutamine accelerated cell growth in RBE cells. Cell proliferation was examined using the CCK-8 assay. Cell number was normalized to day 0. p-values were calculated using the two-way ANOVA. n=5. ***, p < 0.001. H. The expression level of MYC in glutamine treated RBE was detected by western blot (top) and qRT-PCR (bottom). The expression level was normalized to day 0 in HuCCT1. MYC was higher expressed in the IDH1 mutation cell line and was accelerated in RBE cells during the late stage. n=5. ***, p < 0.001.

To further investigate the impact of IDH1 mutations on ICC development, we conducted a comparative analysis of gene expression in tumors from the mouse models with mutant or wild-type IDH1 using RNA sequencing. Analysis revealed that seven out of the top ten Reactome pathways, which consisted of genes that exhibited differential expression, were associated with metabolism. One such example is bile acid metabolism (Fig. 4A), which can impact the initiation and progression of bile duct cancer through multiple mechanisms. As we all know, cholestasis, a disruption in bile acid metabolism, is a significant precursor to cholangiocarcinoma. Metabolic pathways were also significantly enriched in the differentially expressed genes between HuCCT1 and RBE cells (Fig. 4B). This indicated that the presence of IDH1 mutation has a significant impact on the metabolic reprogramming of ICC and affects the development of ICC through this specific pathway. Hence, our study will concentrate on examining the alterations in metabolic pathways in ICC caused by IDH1 mutations.

Specifically, KEGG analysis revealed that the enriched pathways of genes that were upregulated in RBE included alanine, aspartate, and glutamate metabolism (Fig. S3E). Furthermore, we have constructed PPI network via the STRING database, integrating the differentially expressed genes which was significantly upregulated in RBE compared with HuCCT1 from our RNA-seq data and participated in metabolic pathways (hsa01100). PPI network identifies tightly interconnected functional modules within the glutaminolysis pathway, confirming the core metabolic rewiring. KEGG enrichment analysis based on this PPI network has also highlighted that IDH1-mutant ICC reprograms several key metabolic pathways. Notably, alanine, aspartate, and glutamate metabolism is among the most enriched pathways (Fig. S3F-G). We compared the gene expression levels related to glutaminolysis between the HCCT1 and RBE. The study demonstrated an elevation in the expression of most glutaminolysis promotion genes in IDH-mutant cells compared to IDH wild-type cells. Moreover, a significant increase in the expression of more genes linked to the glutaminolysis inhibition was observed in HuCCT1 cells (Fig. 4C). qRT-PCR validation confirmed a substantial elevation in the gene expressions of GLS2, GOT2, GLUD2, GCLC, and ASNS in RBE cells compared to HuCCT1 cells (Fig. 4D). In addition, analysis using the TIMER database showed a significant rise in the expression levels of GOT1, GLS, and ASNS genes in patients with IDH mutant ICC or HCC compared to those with IDH wild-type tumors (Fig. 4E). Overall, these findings suggested an enhanced activity of the glutamine metabolism pathway in tumor cells with IDH1 mutations (Fig. 4F).

To explore the impact of glutamine metabolism on cell growth, we analyzed cell growth curves following extended glutamine exposure. The findings indicated that the growth rate of RBE with IDH1 mutation during the initial phase of glutamine treatment (0-3 days) was comparable to that under glutamine deprivation. However, during the later phase of glutamine treatment (5-7 days), the growth rate of RBE progressively increased (Fig. 4G). These findings indicated the critical role of glutamine in facilitating cell proliferation. The oncogene MYC is well-known for its role in increasing the breakdown of glutamine and enhancing glutamine metabolism by activating the transcription of the GLS and SLC1A5 genes 8. Both qRT-PCR and western blot data consistently illustrated that MYC expression in RBE cells initially declined and then steadily increased throughout the entire duration of glutamine treatment (Fig. 4H). We performed GSEA to investigate that glutamine supplementation (2 mM) for 3days significantly enriches the expression of MYC related genes compared to glutamine-free conditions in IDH1-mutant RBE cells (Fig. S3H). Several studies have demonstrated that metformin, a medication targeting MYC, exhibited notable cytotoxic effects in IDH1 mutant chondrosarcoma, but only after 7 days 21. These findings suggested that MYC promotes cell proliferation during the advanced stages of tumor development.

These results demonstrated that in IDH1-mutant ICC, enhanced glutamine metabolism elevates MYC expression, thereby driving tumor proliferation. Consequently, MYC represented a promising novel therapeutic target for IDH1-mutant ICC.

### MYC targeting leads to IDH1 mutant ICC suppression ex vivo cells and allograft mouse models

**Figure 5:**
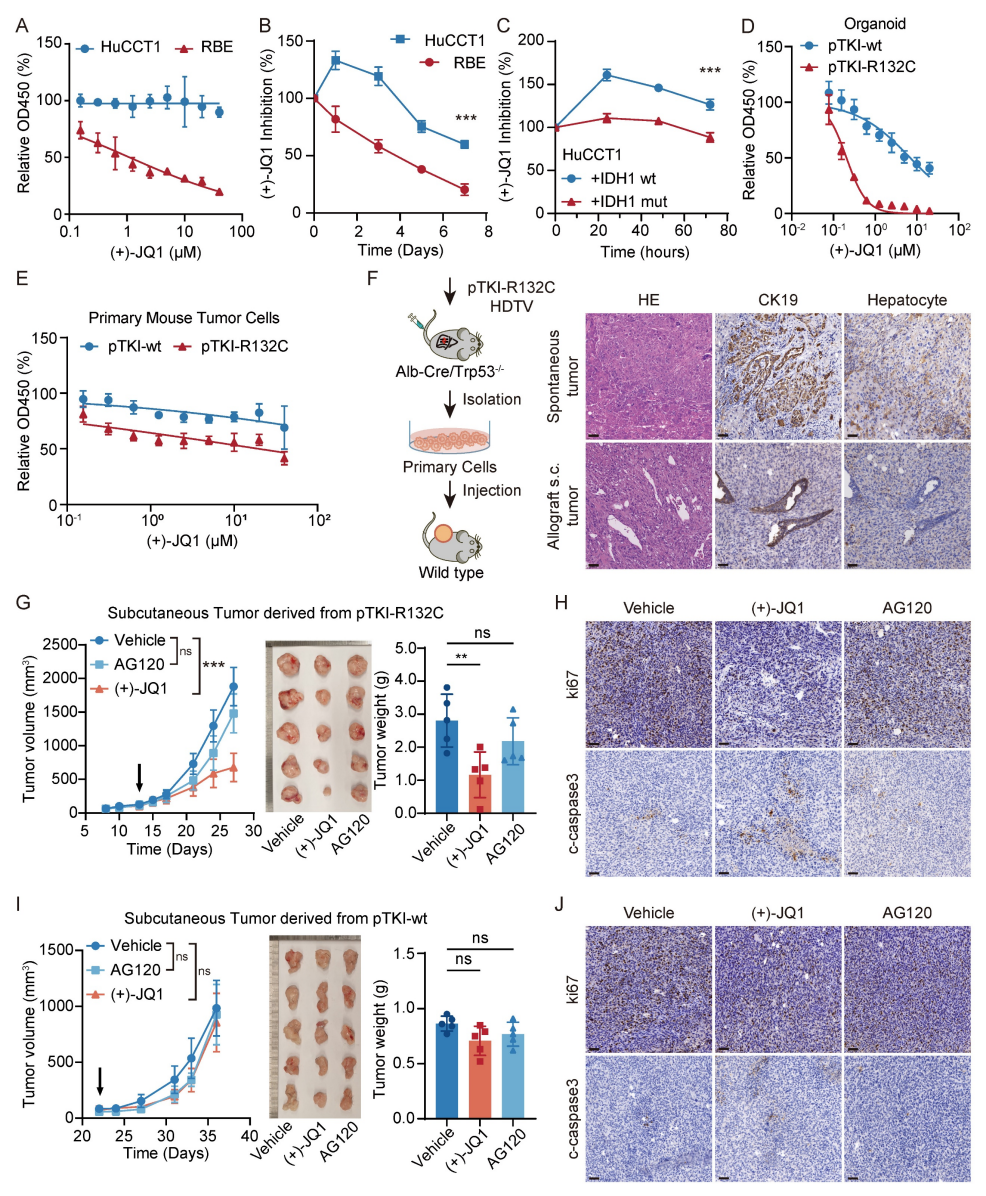
** MYC targeting leads to IDH1 mutant ICC suppression ex vivo cells and allograft mouse models.** A. Dose-response curves of (+)-JQ1 for HuCCT1 and RBE. n=5. B. (+)-JQ1 preferentially suppressed cell growth in RBE cells. Cell proliferation was examined using the CCK-8 assay. Cell number was normalized to day 0. Data represent relative cell viability values, with DMSO-treated cells as control. p-values were calculated using the two-way ANOVA. n=5. ***, p < 0.001. C. IDH1 mutation over-expression significantly promoted the inhibition of (+)-JQ1 in HuCCT1 cells. p-values were calculated using the two-way ANOVA. n=5. ***, p < 0.001. D-E. Dose-response curves of (+)-JQ1 for organoid (D) and primary tumor cells (E) derived from pTKI mouse. n=5. F. Schematic of development of the pTKI-R132C allograft tumor model (left). Representative H&E and IHC staining of spontaneous tumors and subcutaneous ICC allografts (right). Scale bar: 50 μm. G. Growth curves based on tumor volume (left) and representative macroscopic (middle) of tumor tissues from the allogeneic subcutaneous tumor derived from pTKI-R132C mice. The arrow indicates the start of treatment. Rulers in the photo show a minimum unit of mm. Tumors were imaged after the mice were sacrificed after two weeks of treatment. Quantification of tumor burden based on the tumor weight (right). **, p < 0.01. ns, p > 0.05. H. Representative IHC staining of Ki67 and cleaved-caspase3 from s.c. tumor tissues of pTKI-R132 mice treated with vehicle, (+)-JQ1 or AG120. Scale bar: 50 μm. I. Growth curves based on tumor volume (left) and representative macroscopic (middle) of tumor tissues from the allogeneic subcutaneous tumor derived from pTKI-wt mice. The arrow indicates the start of treatment. Rulers in the photo show a minimum unit of mm. Tumors were imaged after the mice were sacrificed after two weeks of treatment. Quantification of tumor burden based on the tumor weight (right). ns, p > 0.05. J. Representative IHC staining of Ki67 and cleaved-caspase3 from s.c. tumor tissues of pTKI-wt mice treated with vehicle, (+)-JQ1 or AG120. Scale bar: 50 μm.

The bromodomain and extra terminal domain (BET) inhibitor (+)-JQ1 binds to the recognition pocket for acetylated lysine residues of BET proteins, predominantly BRD4, effectively downregulating MYC expression in certain tumor types 28. No significant difference in the expression levels of BRD4 were observed between human IDH wt HuCCT1 and IDH1 mutant RBE (Fig. S4A). To investigate the effect of inhibitor (+)-JQ1 on ICC cells, we measured the viability of human ICC cells lines cultured for 24 hours in media with varying concentrations of (+)-JQ1.The dose-response curve analysis revealed that RBE cells, compared to HuCCT1cell line, exhibited heightened sensitivity to (+)-JQ1, as indicated by an IC50 value of 1.110 μM (Fig. 5A). Moreover, time-dependent growth curve analysis showed that (+)-JQ1 treatment provoked strong suppression of RBE tumor cell proliferation, compared to HuCCT1 (Fig. 5B). This inhibitory effect was also observed in mutant IDH1 overexpressing HuCCT1 cells, but not in those with IDH1 wild type (Fig. 5C). Consistently, we found that pTKI-R132C primary mouse tumor cells and organoids displayed notable sensitivity to (+)-JQ1, compared with those derived from pTKI-wt (Fig. 5D-E). Furthermore, GSEA revealed that treatment with the MYC inhibitor (+)-JQ1 leads to significant downregulation of MYC target genes (Fig. S4B). This result provides functional validation that (+)-JQ1 effectively inhibits the MYC-dependent transcriptional network in our IDH1-mutant ICC model.

To investigate the response to pharmacologic inhibition of MYC in vivo, primary cells were subcutaneously transplanted into immunocompetent syngeneic mice to form allograft tumors. Histological assessment confirmed that the engrafted tumors retained the cellular composition of the original primary culture and recapitulated characteristic ICC morphology by H&E and IHC staining (Fig. 5F). Mice bearing allograft tumors ∼100 mm^3^ were administered AG120, (+)-JQ1 or vehicle control. We found that treatment with (+)-JQ1 led to a significant Idh1 mutant tumor suppression compared with vehicle, where AG120 treatment did not affect tumor growth of Idh1 mutant ICC allografts (Fig. 5G). Analysis of pTKI-R132C mouse tumor specimens revealed that (+)-JQ1 treatment markedly decreased tumor cell proliferation (ki67^+^ cells) and induced a degree of cell death (cleaved caspase-3^+^ cells) (Fig. 5H and Fig. S4C-D). In contrast, (+)-JQ1 did not affect tumor cell proliferation and growth of IDH wild-type ICC allografts (Fig. 5I-J and Fig. S4C-D). Taken together, these results demonstrated that MYC targeting has specific efficacy on ICC with IDH mutation.

### MYC targeting prolongs the survival of mice with IDH1-mutant/MYC-amplified ICC

**Figure 6:**
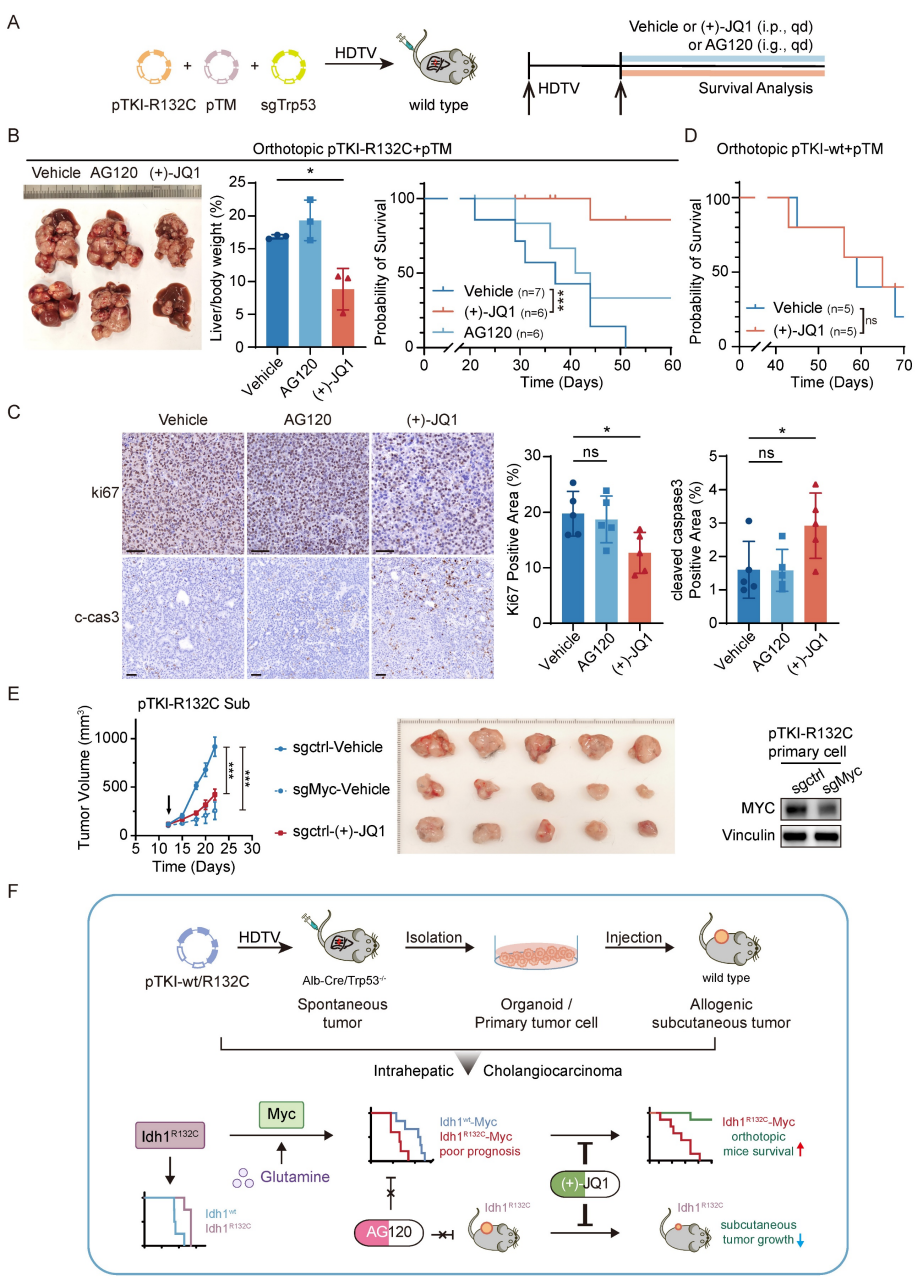
** MYC targeting prolongs the survival of mice with IDH1-mutant/MYC-amplified ICC.** A. Schematic of the experiment. The transposable vector pTM with pTKI-R132C and SB100 vector and sgTrp53 was delivered into Alb-Cre × Trp53^fl/fl^ mice via HDTVi. The mice treated with vehicle or (+)-JQ1 or AG120. B. Representative macroscopic of liver tumor tissues from the pTKI-R132C+pTM mice (left). Quantification of tumor burden based on the liver weight to body weight ratio (middle). Survival graph of the pTKI-R132C+pTM mice (right, vehicle: n=7, other groups: n=6) with different treatments. A log-rank test was used to calculate p-values. *, p < 0.05. ***, p < 0.001. C. Representative IHC images and quantification of area performed by Ki67 and cleaved caspase3 immunohistochemistry on liver tumor tissues of mice treated with vehicle or (+)-JQ1 or AG120. n=5. Scale bar: 50 μm. *, p < 0.05. ns, p > 0.05. D. Survival graph of the pTKI-wt+pTM mice (n=5) with different treatments. A log-rank test was used to calculate p-values. ns, p > 0.05. E. Growth curves based on tumor volume (left) and representative macroscopic (middle) of tumor tissues from the subcutaneous tumor derived from pTKI-R132C primary cells which were knockout Myc via CRISPR/Cas9. The arrow indicates the start of treatment. The expression of Myc in edited cells was detected via western blotting (right). Rulers in the photo show a minimum unit of mm. ***, p < 0.001. F. Schematic of the preclinical model construction process for IDH1 mutation ICC and the mechanism through which MYC overexpression reverses the correlation between IDH1 mutation and favorable outcomes and confers IDH1 mutant ICC marked sensitivity to the BET inhibitor (+)-JQ1.

To evaluate the therapeutic efficacy of AG120 or (+)-JQ1 on orthotopic IDH1-mutant/MYC-amplified ICC mouse models, pTM+pTKI-R132C mice were administered with AG120 via oral gavage or (+)-JQ1 via intraperitoneal injection (Fig. 6A). The results demonstrated that AG120 failed to elicit significant effects on tumor progression or improve overall survival, whereas (+)-JQ1 treatment resulted in a pronounced reduction in tumor burden, as evidenced by the liver-to-body weight ratio, compared to vehicle-treated controls (Fig. 6B). This tumor restriction led to a marked extension in median survival time, suggesting a robust therapeutic benefit of (+)-JQ1 in this genetically defined ICC subtype (Fig. 6B).

Immunohistochemical assessment of tumor tissues revealed a concomitant reduction in Ki67-positive proliferating cells and an increase in cleaved caspase-3 expression, suggesting that (+)-JQ1 exerts its antitumor effects through both antiproliferative and proapoptotic mechanisms (Fig. 6C). Notably, (+)-JQ1 administration did not confer a survival benefit in pTM+pTKI- wt mice (Fig. 6D), underscoring the selective efficacy of this compound in IDH1-mutant ICC models. These results indicated that (+)-JQ1 selectively and effectively suppressed the development and progression of tumors in IDH1 mutant ICC mouse models, highlighting the potential of (+)-JQ1 as a precision therapeutic agent for IDH1-mutant ICC.

To test whether the anti-tumor effects of JQ1 are mediated specifically through MYC inhibition, we generated MYC-knockout PTKI-R132C cells using the CRISPR-Cas9 system and subcutaneously transplanted them, along with control cells, into C57BL/6 mice. The results demonstrated that tumors bearing the sgMyc cells grew significantly slower than those from sgCtrl cells. This genetic ablation of MYC effectively phenocopies the anti-tumor effect of (+)-JQ1 treatment observed in our model (Fig. 6E). This experiment provides direct genetic evidence that the therapeutic efficacy of (+)-JQ1 in IDH1-mutant ICC is mediated through inhibition of the MYC pathway, confirming that MYC is the critical downstream target responsible for the observed anti-tumor activity.

## Discussion

The genomic landscape in ICC is very heterogeneous with less single mutation identified as sufficient to drive tumor onset, necessitating the cooperation of multiple driver genes to initiate the tumor development process 29-30. IDH1 mutations frequently co-occur with activating alterations in the RTK-RAS-MEK pathway, most commonly KRAS mutations 31-33. Previous studies demonstrate that the combination of KRAS^G12D^ and IDH1^R132C^ mutations confers a more aggressive tumor phenotype 15,34. This co-mutation drives tumorigenesis through a synergistic rewiring of cellular metabolism and epigenetic regulation. Based on this combination thereof, we develop orthotopic ICC mouse models by integrating transposon-based Kras and Idh1 mutations into hepatocyte genome of liver conditional Trp53 knockout mice or collaborated with CRISPR-sgTrp53 plasmid into liver tissue of wild type mice. Thus, cooperation of Kras and Idh1 mutations can induce tumor formation in both Trp53 germline and somatic mutation context. Histopathological evaluation confirmed tumors exhibited characteristic ICC features, including positive biliary epithelial markers and IDH1 mutation-associated hypermethylation due to 2-hydroxyglutarate (2-HG) accumulation, which better recapitulated the pathological features of human ICC. A corresponding IDH1 wild-type control model was generated through parallel plasmid modification. In clinical, patients with IDH1 mutant solid tumors demonstrated a robust association with a favorable prognosis, compared to those with IDH1 wild type. Notably, our established IDH1-mutant mice exhibited significantly prolonged median survival compared to IDH1 wild-type counterparts, consistent with clinical observations.

The interplay between IDH1 mutation and MYC oncogene signaling has been extensively investigated across multiple malignancies 35-36. Initial mechanistic studies in leukemia models demonstrated that R-2-hydroxyglutarate (R-2HG), the oncometabolite produced by mutant IDH1, significantly suppresses MYC expression. This occurs through R-2HG-mediated inhibition of fat mass and obesity-associated protein (FTO) demethylase activity, resulting in increased m6A RNA methylation of MYC transcripts and subsequent proteasomal degradation, ultimately impairing leukemic cell proliferation 35. Recent studies have identified a substantial increase in CCDC26 levels in IDH mutant gliomas, accompanied by a marked upregulation of MYC expression 36. Our current findings in ICC models demonstrate that MYC amplification specifically accelerates tumorigenesis in IDH1-mutant murine models. We propose a dual mechanistic hypothesis that MYC mediated functional manifestation of IDH1 mutations: (1) MYC may regulate metabolic pathways that potentiate IDH1-mutant phenotypes, including enhanced 2-HG accumulation, and (2) MYC likely synergizes with mutant IDH1 to amplify downstream oncogenic signaling cascades. This cooperative effect between mutant IDH1 and MYC may create a feed-forward loop that drives more aggressive tumor formation.

Prior to its application in ICC, the IDH1 inhibitor ivosidenib (AG120) demonstrated significant clinical efficacy in acute myeloid leukemia (AML). However, results from the ClarIDHy phase III clinical trial 17 revealed that though ivosidenib-treated ICC patients showed prolonged median overall survival compared to placebo controls, this improvement did not reach statistical significance. Consistent with these clinical observations, our preclinical studies demonstrated limited therapeutic efficacy of AG120 in both in vivo and in vitro ICC models. Importantly, we identified that concurrent IDH1 mutation and MYC amplification confers resistance to AG120 treatment. We propose that this resistance mechanism may involve MYC-driven epigenetic reprogramming through profound DNA methylation alterations that persist despite IDH1 inhibition. Such stable epigenetic modifications may maintain oncogenic phenotypes even after 2-HG reduction by AG120, ultimately leading to therapeutic failure. These findings highlight the importance of comprehensive molecular profiling in clinical practice, suggesting that dual assessment of IDH1 mutation status and MYC expression levels could improve treatment stratification for IDH1-mutant ICC patients. Furthermore, we seek to investigate novel therapeutic strategies for patient subgroups exhibiting both IDH1 mutations and MYC amplification concurrently.

Tumor cells undergo extensive metabolic reprogramming to adapt to the nutrient-depleted and waste-accumulated tumor microenvironment (TME). In IDH1/2-mutant tumors, glutamine catabolism serves as a primary source of α-ketoglutarate (α-KG), creating a potential therapeutic vulnerability 11. This metabolic dependence has spurred clinical investigations, including Phase I trials of the glutaminase inhibitor CB-839 and Phase IB/II trials combining metformin and chloroquine for IDH1/2-mutant solid tumors 22. However, the relationship between IDH1/2 mutations and glutamine dependence is context-dependent, as evidenced by studies showing glutamine reliance in high-grade chondrosarcoma independent of IDH status 21. Our work demonstrates that IDH1-mutant ICC cells exhibit heightened glutamine metabolic pathway activity, with significantly elevated expression of pathway-related genes compared to IDH1-wild-type counterparts. Notably, we observed high MYC expression in IDH1-mutant ICC cells under glutamine-replete conditions, correlating with increased proliferation rates. Given MYC's established role in promoting glutaminolysis through transcriptional activation of *GLS* and *SLC1A5* 8, our findings indicate a feedforward regulatory loop between glutamine metabolism and MYC abundance in IDH1-mutant ICC. We propose a mechanistic model whereby glutamine-derived metabolites facilitate MYC accumulation at promoter/enhancer regions and recruit the epigenetic reader BRD4, thereby amplifying oncogenic transcriptional programs. Supporting this hypothesis, genomic analyses have revealed elevated MYC expression in IDH1-mutant low-grade gliomas (LGG) and glioblastomas (GBM). Similarly, our data demonstrate that IDH1-mutant ICC tumors exhibit increased MYC abundance. These results suggested that targeting MYC could be a crucial therapeutic strategy for solid tumors with IDH1 mutations.

Previous studies have established that the BET inhibitor (+)-JQ1 exerts anti-tumor effects in cholangiocarcinoma (CCA) through multiple mechanisms, including growth inhibition, induction of DNA damage and apoptosis, and suppression of c-Myc and its downstream transcriptional targets involved in cell cycle regulation and DNA repair 37. Our current study demonstrated that (+)-JQ1 exhibits significant anti-tumor activity with notable selectivity for IDH1-mutant ICC in vitro and in vivo models. The molecular basis for this genotype-selective response appears to stem from IDH1 mutation-induced epigenetic and transcriptional reprogramming. We hypothesize that the characteristic 2-hydroxyglutarate (2-HG)-driven hypermethylation phenotype associated with IDH1 mutations creates a unique dependency on MYC-regulated transcriptional programs, thereby increasing cellular vulnerability to BET inhibition. This mechanistic framework is supported by recent findings demonstrating MYC pathway activation in other IDH1-mutant malignancies. Thus, we identified (+)-JQ1 as a promising targeted therapeutic strategy for the IDH1-mutant ICC subset.

In summary, our study establishes novel mouse models that recapitulated the molecular and pathological features of human IDH1-mutant ICC, providing valuable preclinical platforms for therapeutic development. Through integrated in vitro and in vivo analyses, we demonstrate that MYC mediated functional manifestation of IDH1 mutant, leading to worse prognosis. MYC abundance was related to its dependence on glutamine metabolism and conferred resistance to IDH inhibitors. The inhibitor (+)-JQ1 showed selective efficacy against IDH1-mutant ICC. The findings of this study offer guidance for therapeutic decisions in IDH1-mutant ICC, suggesting that the preclinical efficacy of Myc targeting may offer enhanced therapeutic benefits for patients with IDH1 mutant ICC.

## Conclusion

In conclusion, this study identified MYC as both a driver of tumorigenesis in IDH1-mutant ICC and a key therapeutic target for this molecular subtype. The role of MYC offers new insights into that the concurrent detection of MYC amplification in clinical practice could guide therapeutic decisions in IDH1-mutant ICC. The MYC inhibitor (+)-JQ1 may offer enhanced therapeutic benefits specifically for patients with IDH1-mutant/MYC-amplified ICC, offering a potential precision therapy for this patient subset.

## Supplementary Material

Supplementary figures and tables.

## Data Availability

The data used to support the findings of this study are available from the corresponding author upon reasonable request.
