# Supplementary material for: MYC-Mediated Functional Manifestation of IDH1 Mutations in Intrahepatic Cholangiocarcinoma Confers Sensitivity to (+)-JQ1
Source: Int J Biol Sci. 2026 Jan 30;22(5):2247–64. doi: 10.7150/ijbs.123085 (PMC12965060; doi:10.7150/ijbs.123085)
Supplement: Supplementary file 1 — Supplementary figures and tables. [file ijbsv22p2247s1.pdf]

Supplementary materials

Supplementary Figure

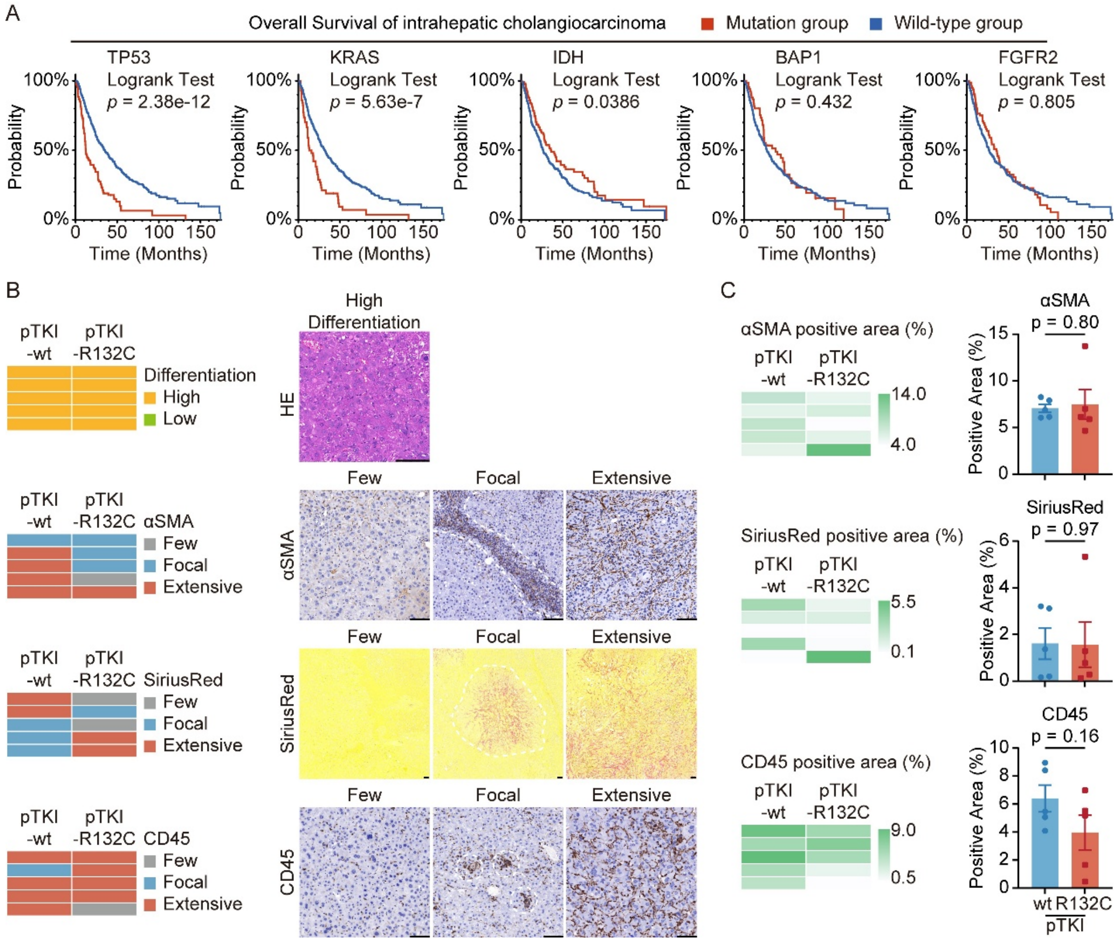

Figure S1

A. Overall survival in intrahepatic cholangiocarcinoma patients with or without the high-frequency genomic alterations in the clinical cohort. Kaplan–Meier curves were analyzed using the log-rank.

B. The bar diagram and representative picture show the differentiation status of each spontaneous tumor from pTKI-wt/R132C mice based on HE staining and the spatial distribution characteristics of representative cells based on IHC staining or Sirus red staining. Scale bar: 100 μm. n = 5 mice per cohort as indicated by bars. Scale bar: 100 μm.

C. The proportion of area performed by α-SMA and CD45 immunohistochemistry or Sirus red staining was quantified and illustrated by the bar chart. n = 5 mice per cohort as indicated by bars.

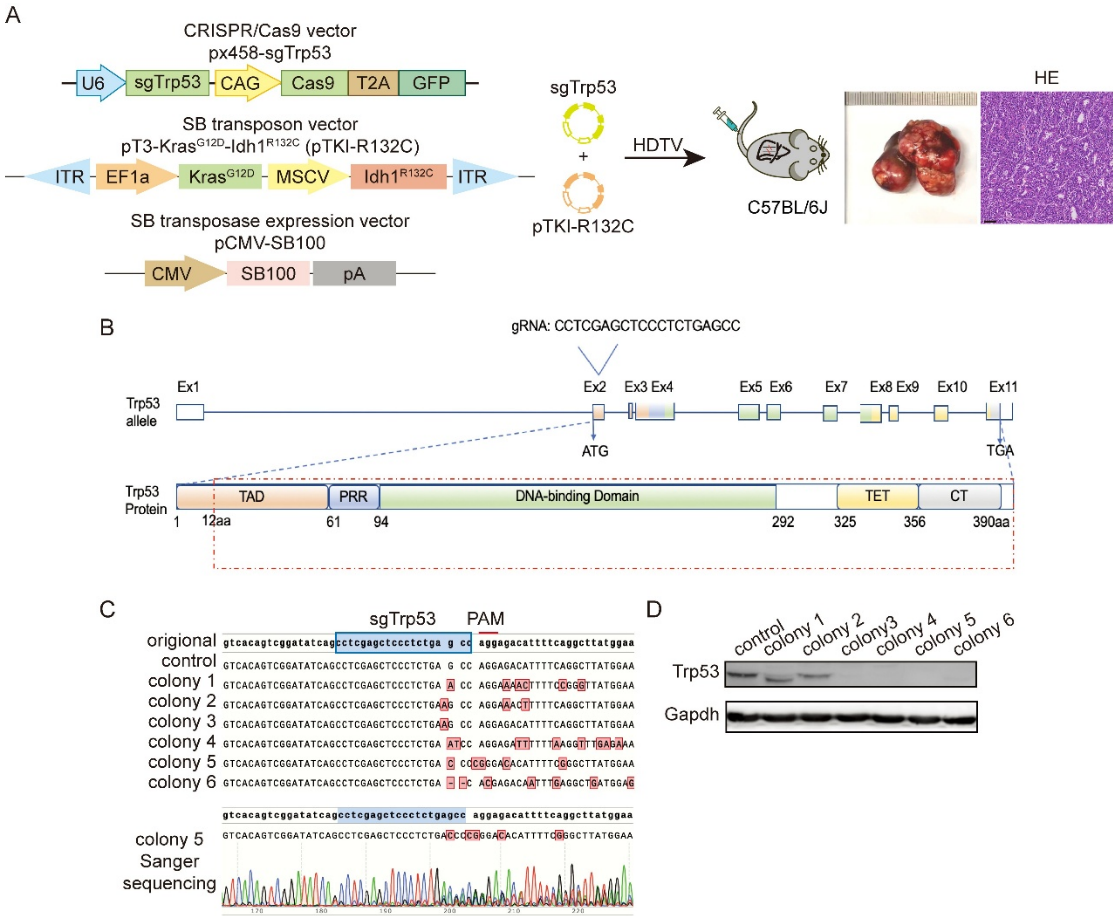

Figure S2

A. Schematic of the mouse model. The CRISPR/Cas9 vector PX458-sgTrp53 with pTKI and SB100 vector was delivered into wild-type mice via HDTVi. Scale bar: 50 μm.

B. Schematic diagram of the Trp53 gene sequence in mice.

C. The sequence of the Trp53 gene in cells after knockout with px458-sgTrp53.

D. The expression of Trp53 protein in cells after knockout with px458-sgTrp53 was detected by western blot.

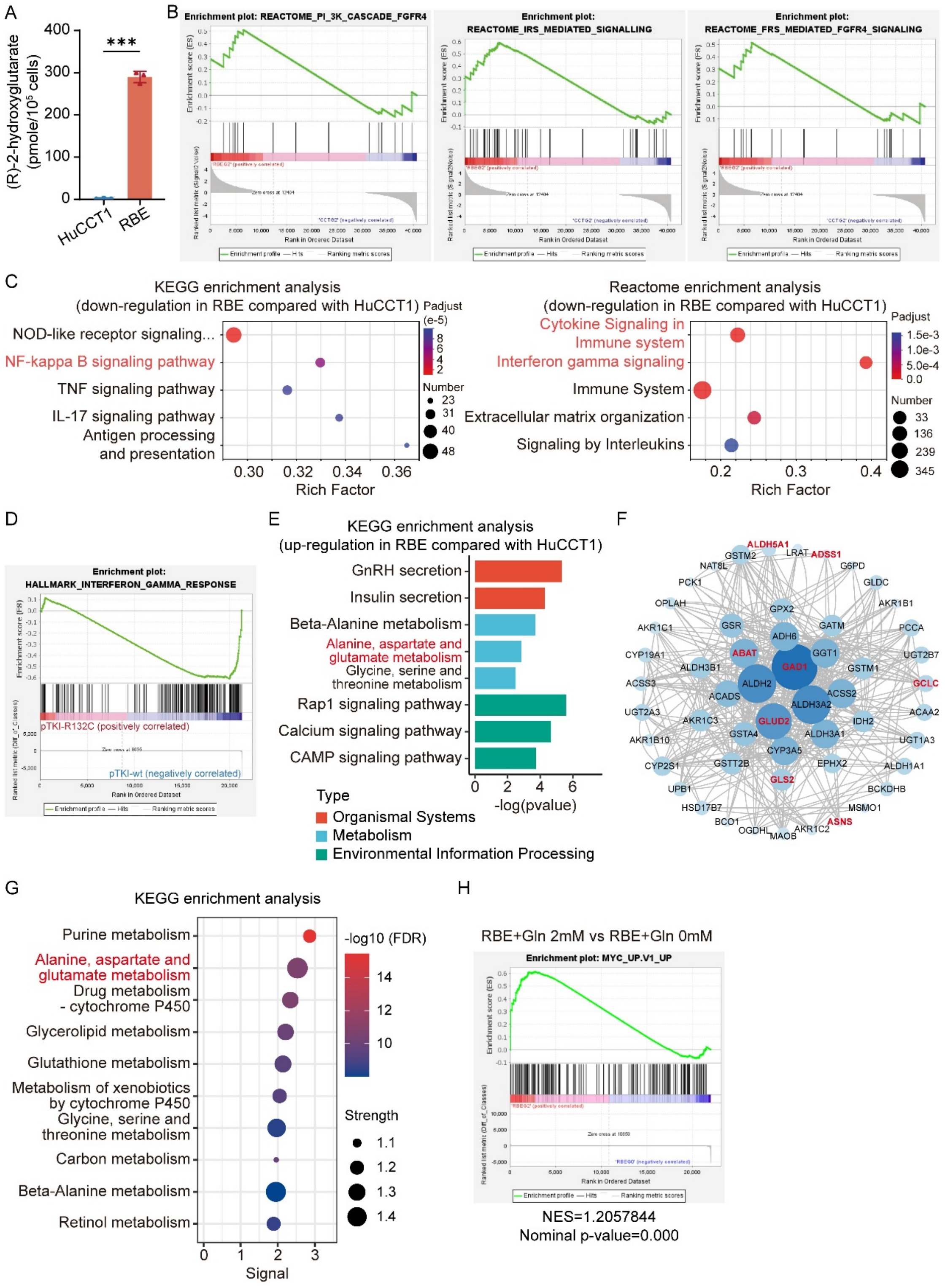

Figure S3

A. Concentration of (R)-2HG in cell lines detected by enzymatic assay kits. n=3. ***, $p < 0.001$.

B. GESA was performed to analyze the gene sets with significantly increased expression in RBE compared with HuCCT1. Significant enrichment was considered as $q < 0.25$.

C. KEGG enrichment analysis and Reactome enrichment analysis were performed for differential genes with significantly lower expression in RBE compared with HuCCT1 obtained by RNA-seq.

D. GESA was performed to analyze the gene sets with significantly increased expression in pTKI-R132C compared with pTKI-wt. Significant enrichment was considered as $q < 0.25$.

E. KEGG enrichment analysis was performed for genes with significantly upregulation in RBE compared with HuCCT1 obtained by RNA-seq. The figure only shows the top 8 pathways excluding those related to human diseases.

F. PPI network via STRING was created with genes included in metabolic pathways (has01100) and with significantly upregulation in RBE compared with HuCCT1 obtained by RNA-seq. The network diagram only shows the genes with a degree greater than 10 that were calculated using Cytoscape. The network highlights genes belonging to the alanine, aspartate, and glutamate metabolism pathway.

G. The dots continuously deepen in color or increase in size as the degree increases. The bubble diagram shows top10 KEGG enrichment pathways in the network.

H. GESA was performed to analyze the gene sets with significantly increased expression in RBE treated with 2 mM glutamine compared with glutamine free. MYC_UP.V1_UP Gene Set includes genes up-regulated in primary epithelial breast cancer cell culture over-expressing MYC.

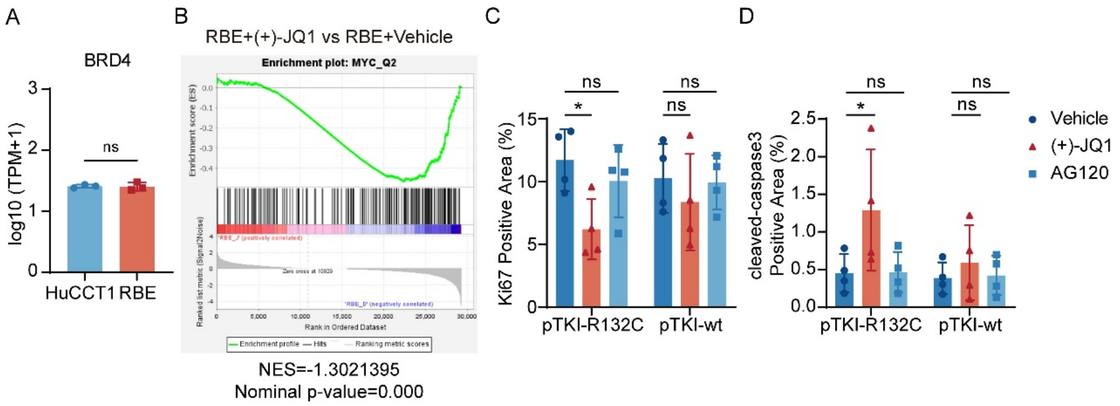

Figure S4

A. RNA sequencing determined differences in BRD4 gene expression between two cell lines. n=3. ns, $p > 0.05$.

B. GESA was performed to analyze the gene sets with significantly depressed expression in RBE treated with (+)-JQ1 compared with vehicle. MYC_Q2 Gene Set includes genes having at least one occurrence of the motif CACGTGS in the regions spanning 4 kb centered on their transcription starting sites [-2kb, +2kb].

C-D. Quantification of area performed by Ki67 and cleaved caspase3 immunohistochemistry on s.c. tumor tissues of pTKI-wt/R132C mice treated with vehicle, (+)-JQ1 or AG120. *, $p < 0.05$. ns, $p > 0.05$.

Supplementary Table 1 qRT-PCR primer

Gene	Primer sequence
MYC	F: 5′-GGATTCTCTGCTCTCCTC-3′<br>R: 5′-CTTGTTCCTCCTCAGAGTC-3′
GLS	F: 5′-TGGTGGCCTCAGGTGAAAAT-3′<br>R: 5′-CCAAGCTAGGTAACAGACCCTGTTT-3′
GLS2	F: 5′-AACGAATCCCTATCCACAAGTTCA-3′<br>R: 5′-GCAGTCCAGTGGCCTTTAGTG-3′
GOT1	F: 5′-GCTGTGCTTCTCGCCTAGTT-3′<br>R: 5′-AAGACTGCACCCCTCCAAC-3′
GOT2	F: 5′-AGGGGAGGGTTGGAATACAT-3′<br>R: 5′-AATGTTTGCCTCTGCCAATC-3′
GLUD1	F: 5′-AGGAATGACACCAGGGTTTG-3′<br>R: 5′-TCAGACTCACCAACAGCAATAC-3′
GLUD2	F: 5′-CACTCTGCCTTGGCATACAC-3′<br>R: 5′-CTCAGGTCCAATCCCAGGTT-3′
GCLC	F: 5′-ATGCCATGGGATTTGGAAT-3′<br>R: 5′-AGATATACTGCAGGCTTGGAATG-3′
ASNS	F: 5′-GCAGTGTCTGAGTGCGATGAA-3′<br>R: 5′-TCTTATCGGCTGCATTCCAAAC-3′
U6	F: 5′-ATTGGAACGATACAGAGAAGAT-3′<br>R: 5′-TTCACGAATTTGCGTGTCAT-3′

Supplementary Table 2 Antibodies

Antibodies	Clone	Cat#	Source
Anti-CK19	EP1580Y	ab52625	Abcam
Anti-Heapatocyte	OCH1E5	ab75677	Abcam
Anti-Ki67	SP6	ab15580	Abcam
Anti-5-Methylcytosine	33D3	39649	Proteintech
Anti-5-Hydroxymethylcytosine	pAb	39792	Proteintech
Anti-CD45	D3F8Q	70257	Cell Signaling Technology
HRP-anti-rabbit IgG		PV-6001	ZSGB
HRP-anti-mouse IgG		PV-6002	ZSGB
Anti-alpha Tubulin		14555-1-AP	Proteintech
Anti-Vinculin		26520-1-AP	Proteintech
Anti-c-Myc	Y69	ab32072	Abcam
anti-mouse IgG, HRP-linked antibodies		7076	Cell Signaling Technology
anti-rabbit IgG, HRP-linked antibodies		7074	Cell Signaling Technology
